# Structured surface feature classification on DED-printed parts. Exemplary data and comprehensive influences listing

**DOI:** 10.1016/j.dib.2025.112427

**Published:** 2025-12-31

**Authors:** Jonny Kaars, Jonas Hensel

**Affiliations:** Chemnitz University of Technology, Strasse der Nationen 62, 09111 Chemnitz

**Keywords:** Directed energy deposition, Features, Defects, Surface, Quality

## Abstract

Directed Energy Deposition is a proven technology for the economic production of medium to large sized metal structures. The process results in a characteristic surface structure composed of the molten layers. It is evident that, the surface structure impairs the strength of the workpiece during static and cyclic loading. Consequently, surface topology evaluation is crucial in the assessment of workpieces manufactured by Directed Energy Deposition. In this work, comprehensive examples of structured feature assessment of real-world workpieces are collected. The examples supplement tabular technical specification of surface features with visual topology appearances.

Specifications Table

**Table:** 

Subject	Engineering & Materials science
Specific subject area	Structured Surface Topology Assessment of Directed Energy Deposition Workpieces based on Features, Comprehensive Cause-Effect relation diagrams composing the origins and influence factors of the features
Type of data	Images, Analyzed and annotated Graphs, Processed.
Data collection	Pre-existing workpieces from various real-world applications and feasibility studies were investigated. The samples were visually inspected and peculiar features identified, listed, measured and photographed. After sorting of features, structured feature labels according to the feature table nomenclature from the related research article where assigned. Influence factors on the surface have been compiled based on experiences in own research works, discussion with industry partners and from ongoing research projects. Samples #24 … #30 courtesy of GEFERTEC, Berlin Materials and Methods used: Comprehensive Sample Collection at the authors institute, industrial samples, Various digital photo equipment, Editing Software GIMP 3.0.4, Annotation in Software Inkscape 1.4.2, Draw.io for Schematic Figures.
Data source location	Chemnitz University of Technology, Chair of Welding Engineering.
Data accessibility	Repository name: DED-Surface Feature Sample Catalogue Data identification number: (10.17632/gp2vrt26cx) Direct URL to data: https://data.mendeley.com/datasets/gp2vrt26cx Repository name: Influence factors on the surface structure of DED-workpieces Data identification number: (10.17632/v759h5sdjs) Direct URL to data: https://data.mendeley.com/datasets/v759h5sdjs Instructions for accessing these data: Browse the URL provided and review the files presented
Related research article	‘Structured Approach towards Surface Feature Classification on DED-printed Parts’ Journal of Materials and Design, 2025 https://doi.org/10.1016/j.matdes.2025.114855 https://www.sciencedirect.com/science/article/pii/S0264127525012754; [1].

## 1 Value of the Data

• The value of the data results from two aspects
  ○ Connecting structured feature descriptions with technical nomenclatures with a comprehensive selection of human-understandable real world examples, covering most cases of features. The data provides a fundamental basis for future standardization work on features and derivated defects on Directed Energy Workpieces.
  ○ Proving applicability of structured feature labelling according to the structured feature table on a wide selection of real world examples.
• The data can be readily used by comparing own results with examples given here, supplementing decisions during classification of ambiguous features.
• Influence factors on the surface topology, i.e. surface features, are comprehensively compiled in this work, forming a basis for future standardization work as well as defect-cause analysis.
• Fields of Application: Structured features and their causes are very relevant to industrial uses of large load bearing components made by directed energy deposition in regulated industries, such as pressure vessels, steel constructions, marine and naval vessels, railroad and many more.

## 2 Background

Additive Manufacturing of metal structures using directed energy deposition features many advantages and chances. However, static and cyclic strength of the workpiece are impaired by its characteristic surface structure. So far, no standards exist covering the description of such features. In the related research article, a method for structured, unequivocal and technical description of features is proposed.

The data in this data article acts as a supplementing example catalogue. Examples have been assessed and labelled according to the new method and can be readily assessed by the reader. Thus, the comprehensive list of examples connects a very abstract and theoretical concept with real world applications. This enhances understandability of the new concept, proves applicability to real workpieces and thus greatly enhances the value of the research work for the next steps of future work towards standardization.

Without the data, assessment and understanding of the original research works’ outcome would be greatly impaired, while at the same time the amount of data would make the original work too long, it was included therein fully.

## 3 Experimental Design, Materials and Methods

**Fig. 1:**
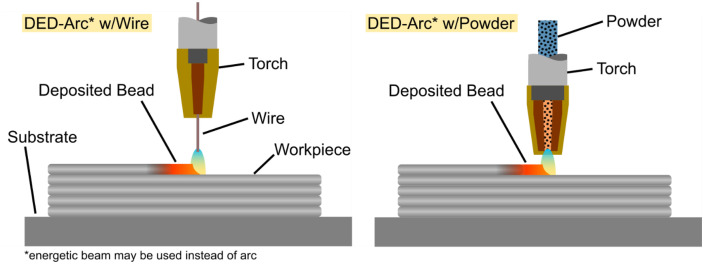
Directed energy deposition process principle.

Pre-existing workpieces from various real-world applications and feasibility studies were investigated. The samples were produced by DED-Arc/M according using either wire or powder-based filler material feed. The process is depicted in Fig. 1. Principally the arc could have been replaced by energetic beams such as laser or electron beam, with laser-based DED, both with wire and powder, being widely used in the industry. The beam processes are mentioned here, because Independently of the energy source the surface features and influences factors listed here apply in the same way. Alloys and process parameters used for the samples are not discussed here to stay within scope of this work.

The samples were visually inspected and peculiar features identified, listed, measured and photographed. After sorting of features, structured feature labels according to the feature table nomenclature from the related research article where assigned. Influence factors on the surface have been compiled based on literature reports, experiences in own research works, discussion with industry partners and from ongoing research projects.

Samples #24 … #30 courtesy of GEFERTEC, Berlin.

Materials and Methods used: Comprehensive Sample Collection at the authors institute, industrial samples, Various digital photo equipment, Editing Software GIMP 3.0.4, Annotation in Software Inkscape 1.4.2, Draw.io for Schematic Figures.

## 4 Data Description

### 4.1 Surface feature example catalogue

The example images are continuously numbered ‘example #i’, with each image providing at least one structured labeling according to the original work imprinted within the image.

**Fig. 2:**
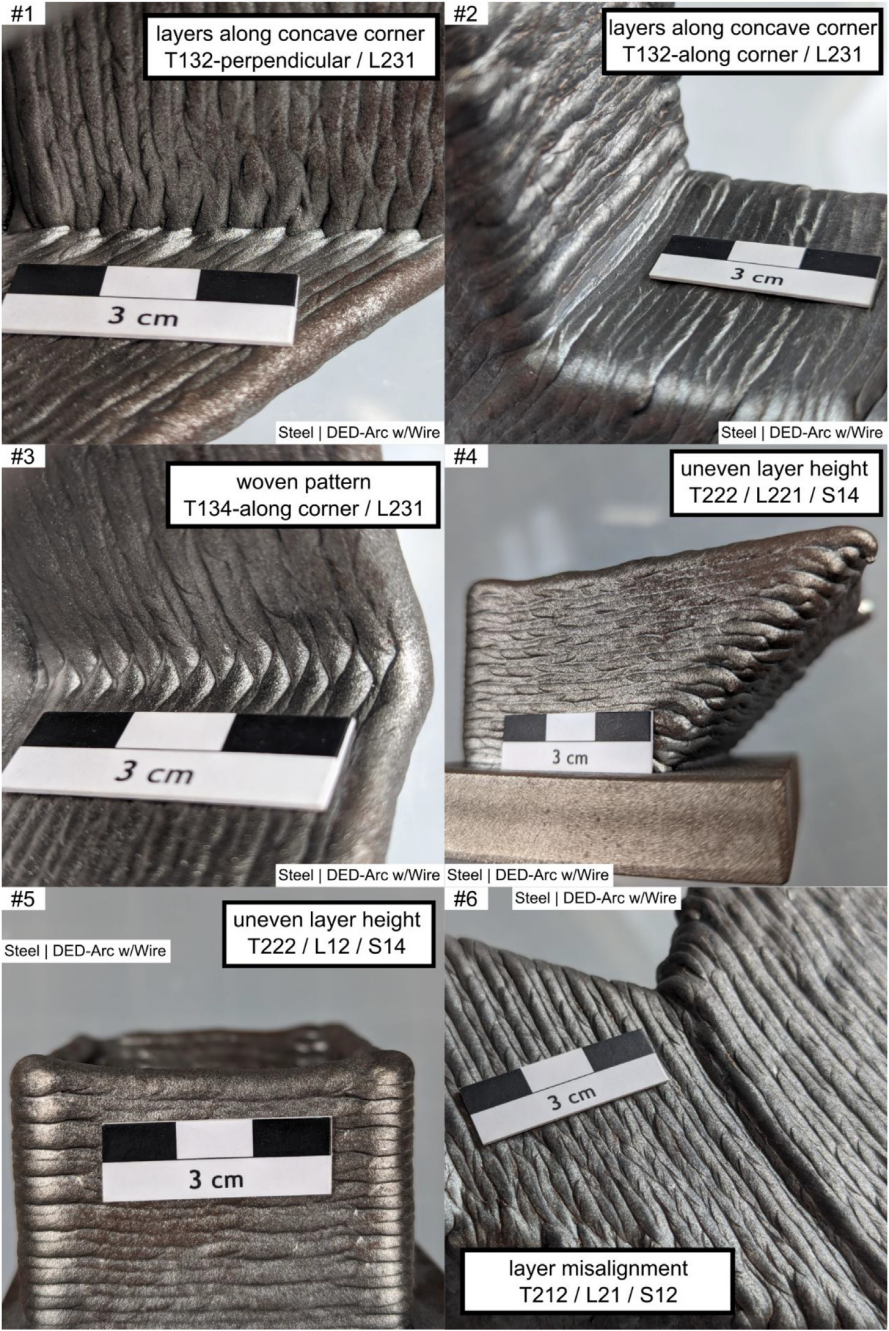
Example #1 ... #6.

**Fig. 3:**
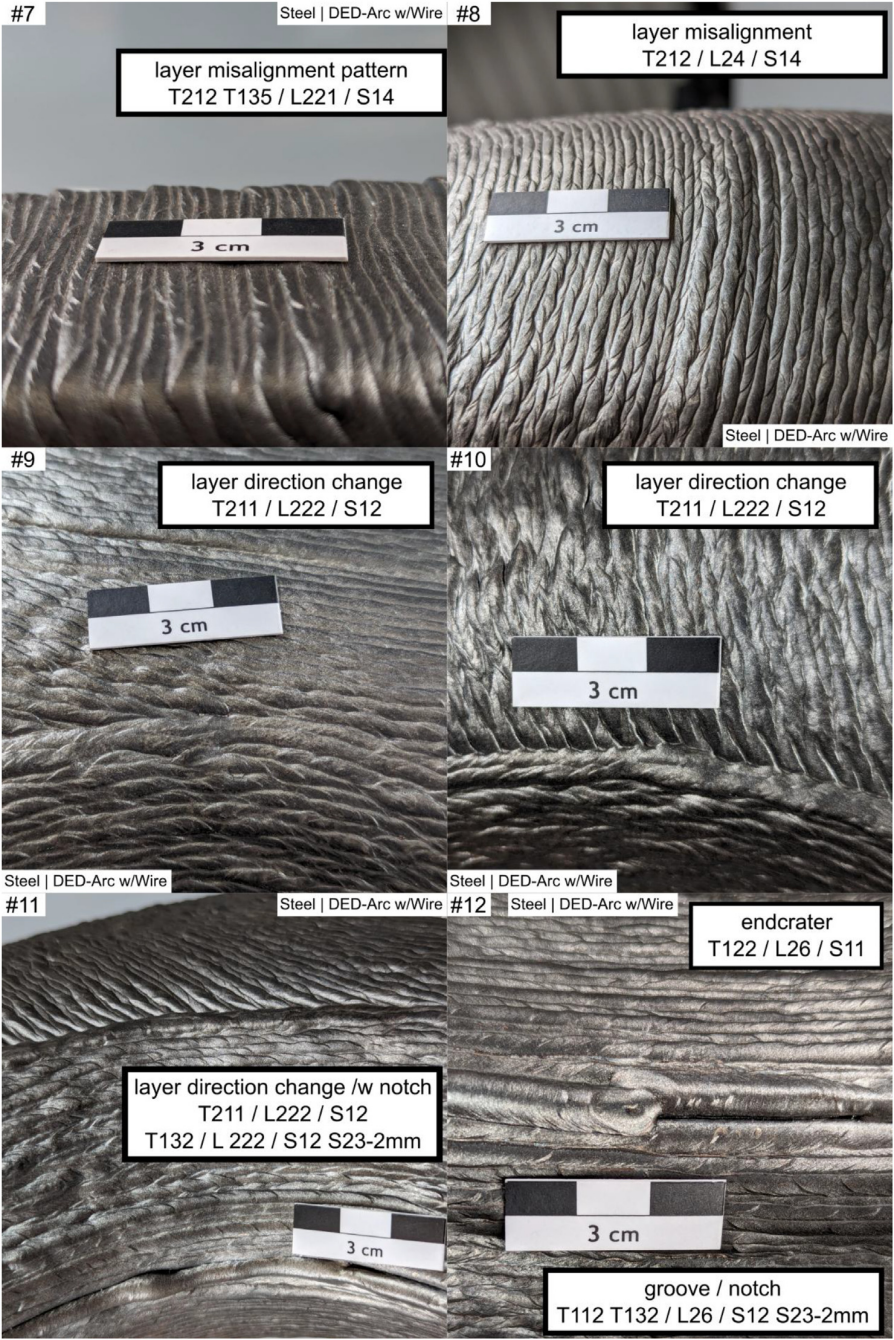
Example #7 ... #12.

**Fig. 4:**
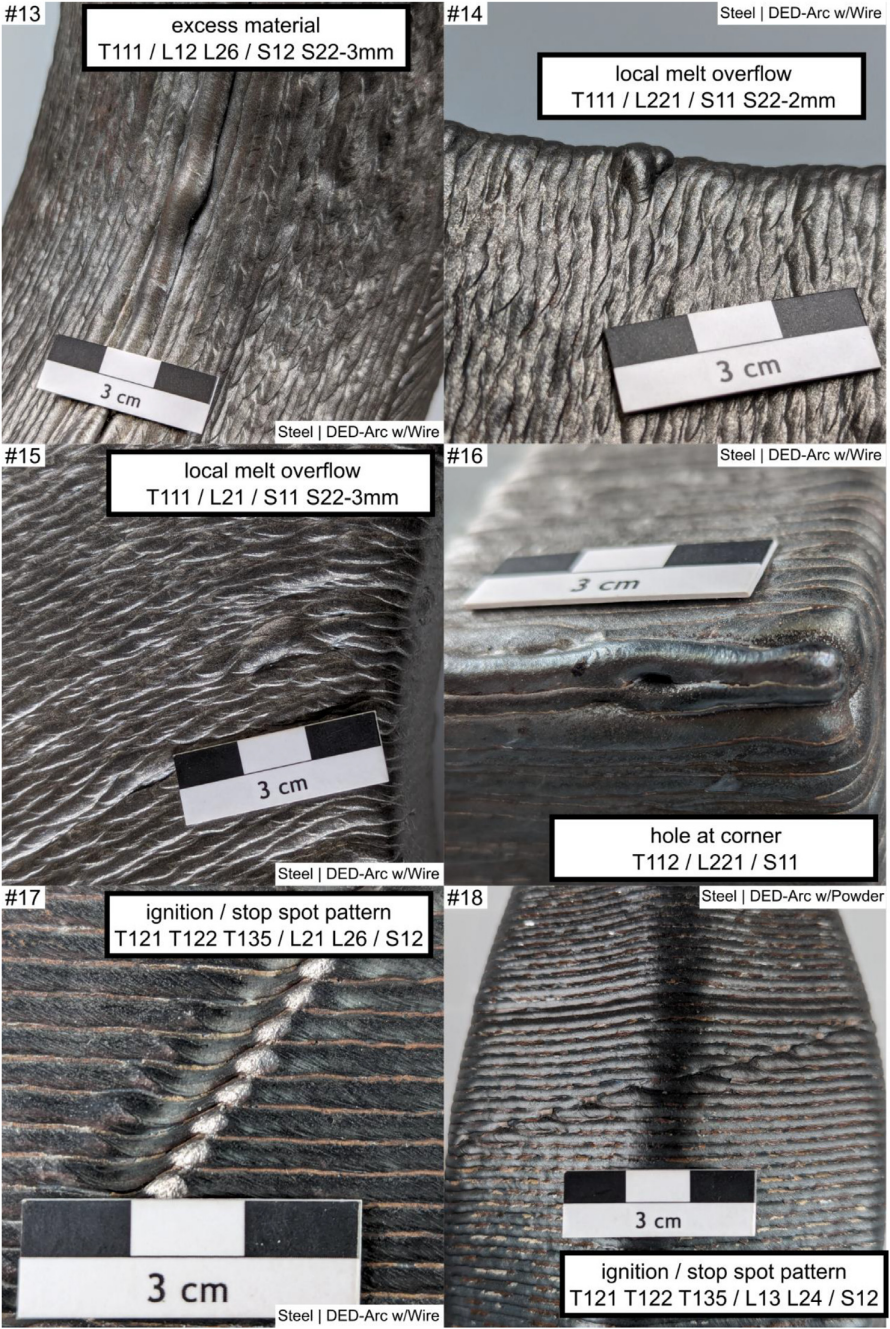
Example #13 ... #18.

**Fig. 5:**
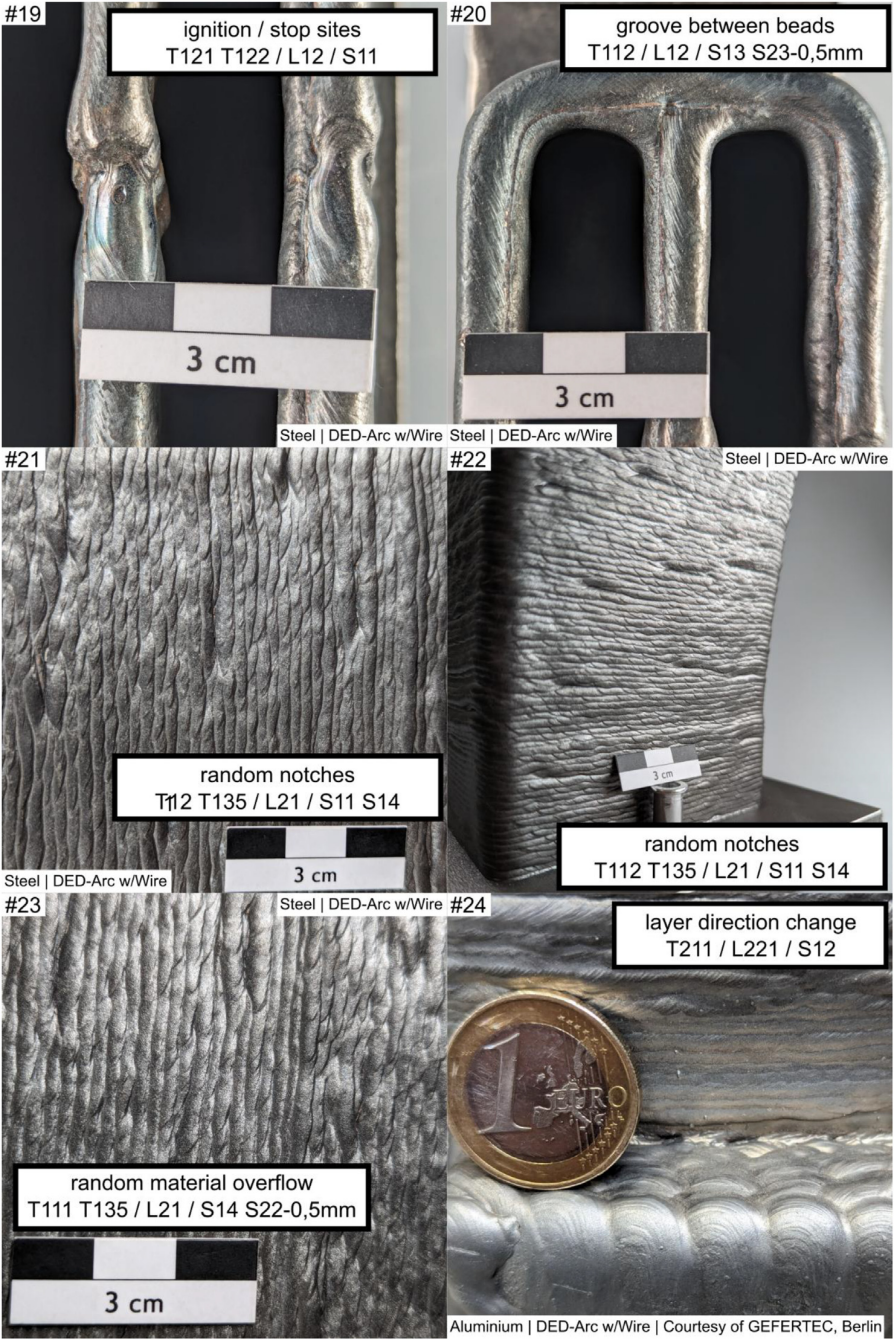
Example #19 ... #24.

**Fig. 6:**
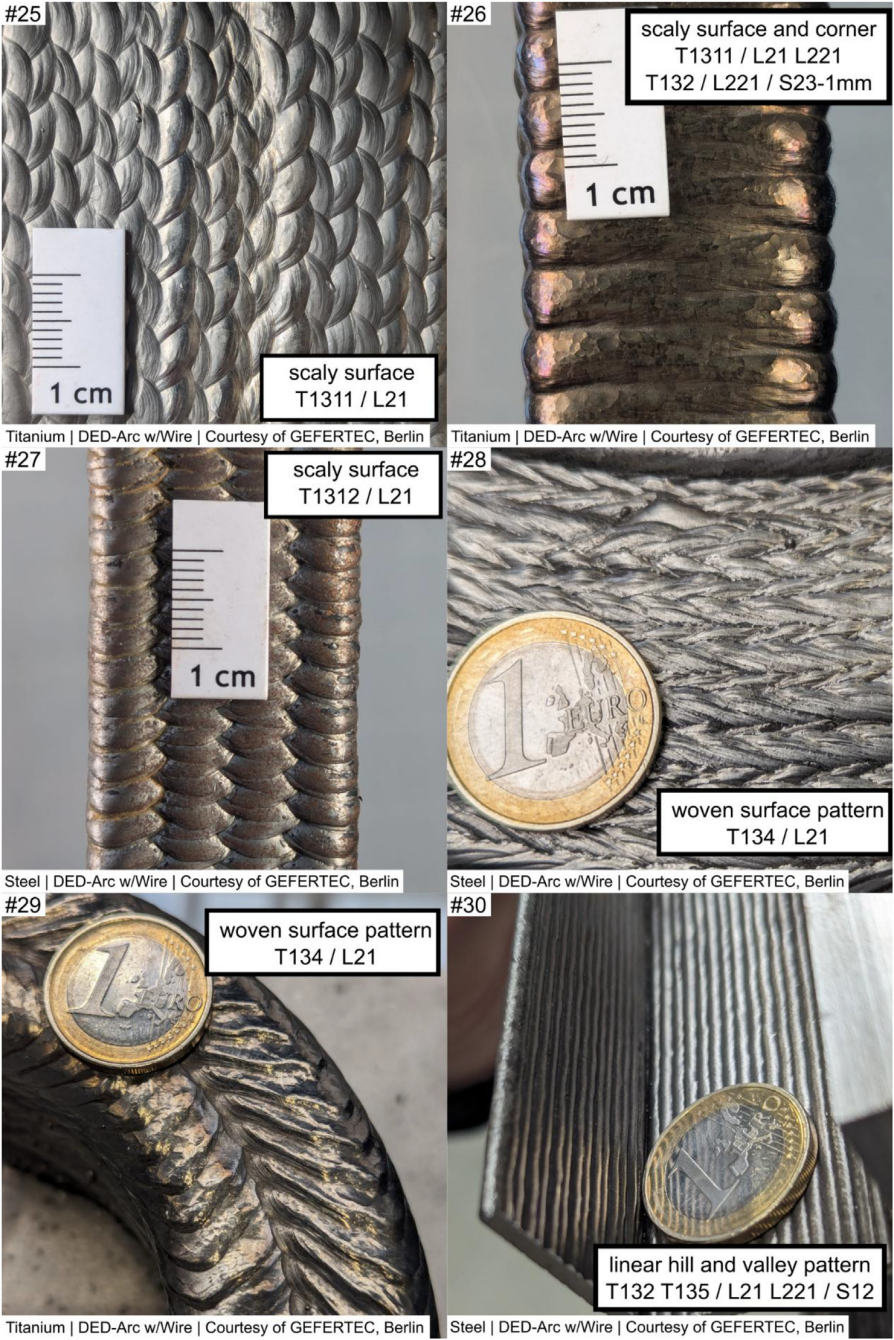
Example #25 ... #30.

The examples are composed as follows, see Fig. 2, Fig. 3, Fig. 4, Fig. 5, Fig. 6. Denotations and brief descriptions to the examples are composed in Table 1, following the examples.

**Table 1:** Designations and descriptions of examples.

**Example #** **Designation**	**Description**
1 layers along concave corner	Layers are oriented perpendicular (T132 - perpendicular) to a sharp concave corner (L231), forming a characteristic notch shape
2 layers along concave corner	Layers are oriented parallel (T132 – along corner) to a sharp concave corner (L231), forming a characteristic notch
3 woven pattern	Layers are oriented perpendicular (L231) to a soft concave corner, forming a characteristic distinct woven pattern (T134 - woven)
4 uneven layer height	Multiple layers (S14) at a uniaxial convex corner (L221) show an uneven layer height (T222), resulting in uneven surface profile
5 uneven layer height	Multiple layers (S14) at a flat area (L12) show an uneven layer height, resulting in uneven height profile of the layers and workpiece (T222)
6 layer misalignment	Working planes of consecutive layers are shifted against each other (T212), causing a line-shaped (S12) distorted geometry in a flat area (L12)
7 layer misalignment pattern	Layers are repeatedly (T135) misaligned (T212), affecting multiple layers (S14) at a convex corner (L221); the example shows concatenation of labels if needed
8 layer misalignment	Multiple (S14) layer misalignment (T212) on a convex cambered surface (L24)
9 layer direction change	On a multiaxially curved surface (L222) the layer orientation changes (T211), resulting in a characteristic linear groove (S12)
10 layer direction change	On a multiaxially curved surface (L222) the layer orientation changes (T211), resulting in a characteristic linear groove (S12); Alternate appearance of example #9
11 layer direction change /w notch	On a multiaxially curved surface (L222) the layer orientation changes (T211), resulting in a characteristic linear groove (S12), the groove (T132 - ‘omitted’) on the surface (L222) is 2 mm deep (S23 - 2 mm)
12 Endcrater; groove / notch	Single (S11) endcrater (T122) on a saddle surface (L26); next to it a groove (T112 T132 - ‘omitted’) of 2 mm dept (S23 - 2 mm)); T112 and T132 are alternate descriptions
13 excess material	Linear-shaped (S12) material overflow (T111) of 3 mm height (S22 - 3 mm) at the last bead (L12) of the workpiece on a saddle surface (L26) of the workpiece
14 local melt overflow	Dot-shaped (S11) material overflow (T111) of 2 mm height (S22 - 2 mm) at a convex corner (L221)
15 local melt overflow	Dot-shaped (S11) material overflow (T111) of 3 mm height (S22 - 2 mm) at a flat area (L21)
16 Hole at corner	Dot-shaped (S11) material deficiency (T112) at a convex corner (L221)
17 ignition / stop pattern	Ignition (T121) and Stop (T122) sites in repeating pattern (T135) at a flat area (L21) shaping a linear groove (S12)
18 ignition / stop pattern	Ignition (T121) and Stop (T122) sites in repeating pattern (T135) between workpiece top and bottom (L13) an a convex cambered surface (L24), shaping a linear groove (S12)
19 ignition / stop sites	Ignition (T121) and Stop (T122) sites at the workpiece top (L12), dot shaped (S11)
20 groove between beads	Material Deficiency (T112) at the workpiece top (L12); planar shape (S13) and 0.5 mm depth (S23 – 0.5 mm)
21 random notches	Dot-shaped (S11) material deficiency (T112) in unspecified repeating pattern (T135) on a flat surface (L21) over multiple layers (S14)
22 random notches	Alternate appearance of example #21
23 random material overflow	material overflow (T111) in unspecified repeating pattern (T135) on a flat surface (L21) over multiple layers (S14), 0.5 mm high (S22 – 0.5 mm)
24 layer direction change	Layer Orientation Change (T211) at a uniaxial convex corner (L221) forming a linear (S12) feature
25 scaly surface	Fishscale-like pattern (T1311) on a mainly flat area (L21)
26 scaly surface and corner	Alternating surface pattern (T1311) on a mainly flat area (L21) and uniaxial convex corner (L221) Alternative Label: Grooves (T132) on uniaxial convex corner (L221), 1 mm deep (S23 – 1 mm)
27 scaly surface	Scales in repeating, in-line pattern (T1312) on a mainly flat area (L21)
28 woven surface pattern	Woven surface pattern (T134) on a mainly flat area (L21)
29 woven surface pattern	Alternate appearance of example #28
30 Linear hill and valley pattern	Linear shaped (S12) grooves (T132) in repeating pattern (T135) on a mainly flat area (L21)

Note: depth measure references shall be stated in workpiece report, usually the Taubin-filtered geometry is used

### 4.2 Influence factors on surface features

The surface features on directed-energy-deposition workpieces are a result of the totality of process influence factors. As number of existing influence factors is huge, it is useful to logically group them. The result, along with brief descriptions, is depicted in the following figures.

**Fig. 7:**
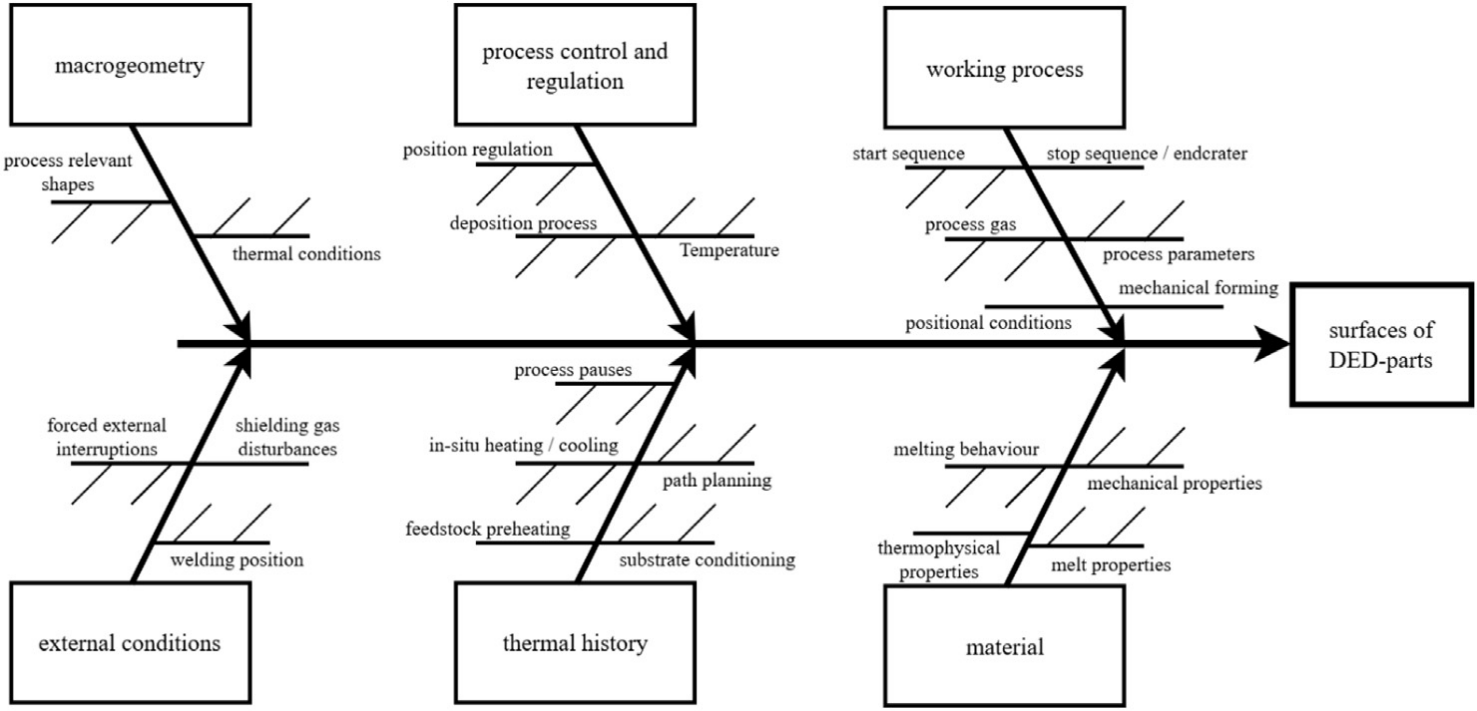
Influence factors overview.

The influences on the surface topology on DED-workpieces stem from six factor groups: Macrogeometry of the workpiece itself, process control and regulation, the working process itself, external conditions, thermal history during workpiece buildup and building material. All conceivable influence factors on the surface can be linked to one of those groups. Fig. 7

**Fig. 8:**
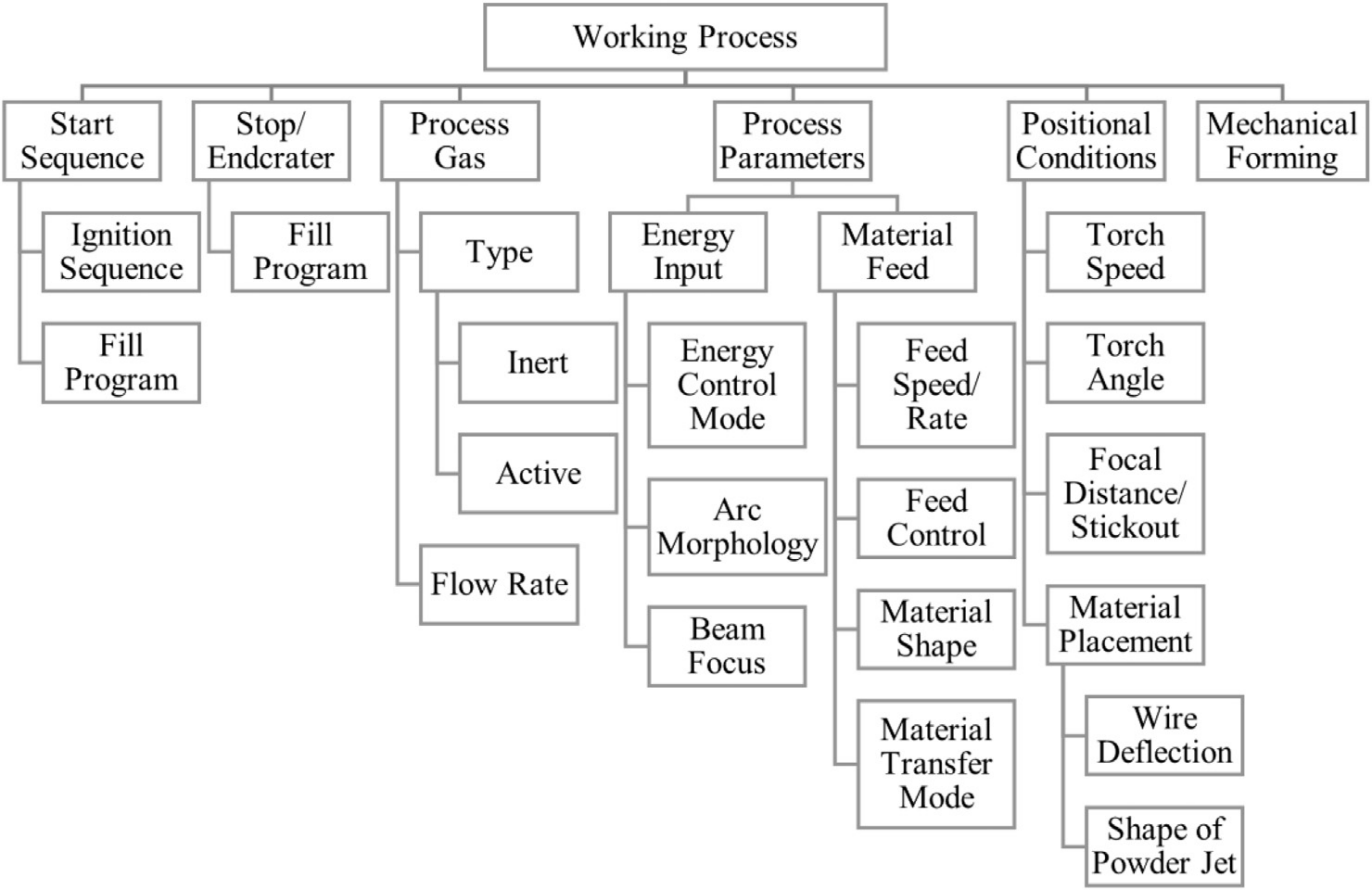
‘Working process’ influence group.

The working process, Figure 8, is one of the most complex influence groups: Methods used for Start- and Stop locations yield specific surfaces at those locations specific for DED. Process gas type and flow rate determine surface appearance by influencing the melt pool´. Arc morphology and energy control or beam focus influence the reaction to irregularities, thus influencing the melt pool. Related are material feed properties, such as feed rate and control, material shape (powder, wire, rod, …) and transfer mode. Together with positional properties of torch and material, the melt pool shape is determined. After solidification, the deposited bead may be reshaped by mechanical forming by a roller, hammering pin or such, reshaping the surface.

**Fig. 9:**
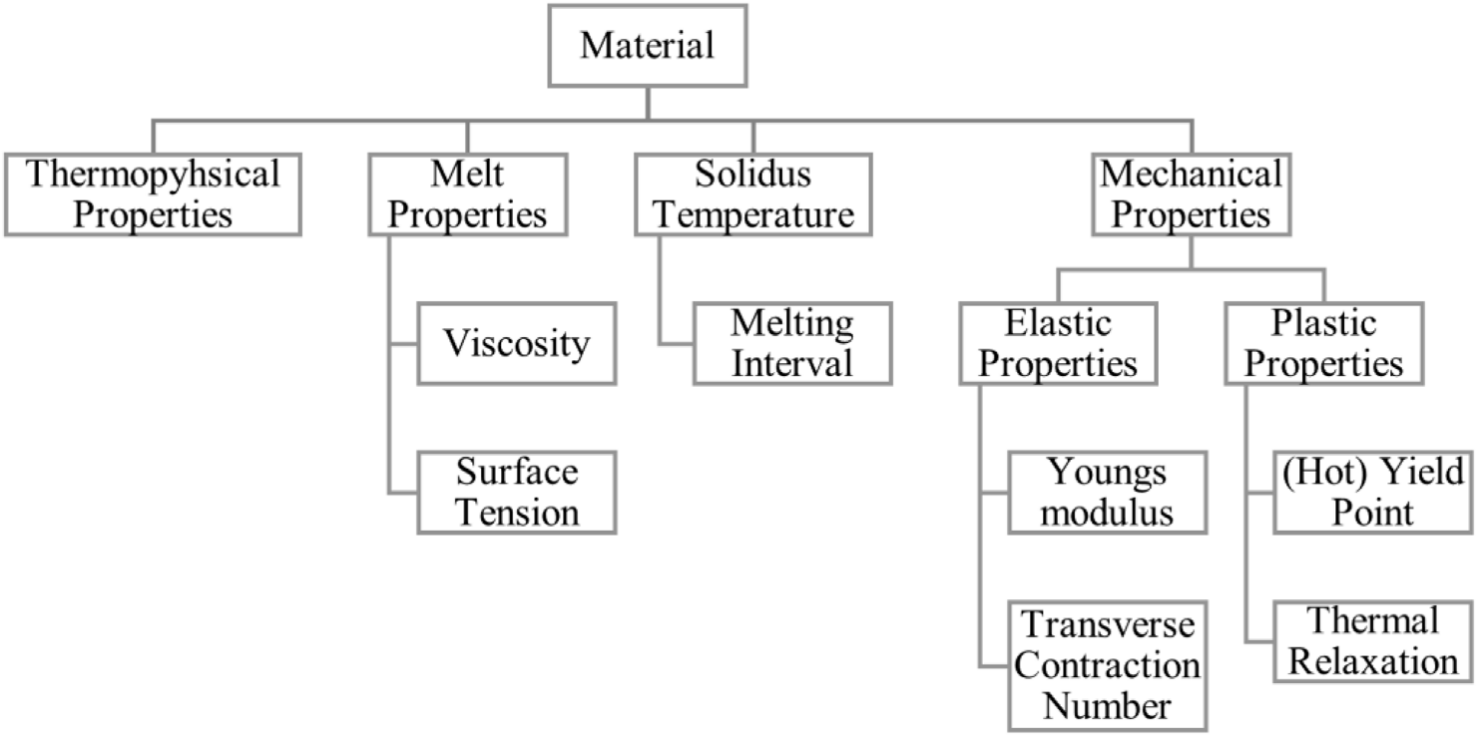
‘Material’ influence group.

The deposited material, Fig. 9, introduces physical prerequisites into the process, most importantly thermophysical properties such as heat conductivity and density, but also melt properties such as viscosity, surface tension and melting interval. After solidification, mechanical properties determine the establishing of thermally induced strain, that is distortion of the workpiece, which again will influence the shape of surface as the workpiece repositions itself below the torch [2].

**Fig. 10:**
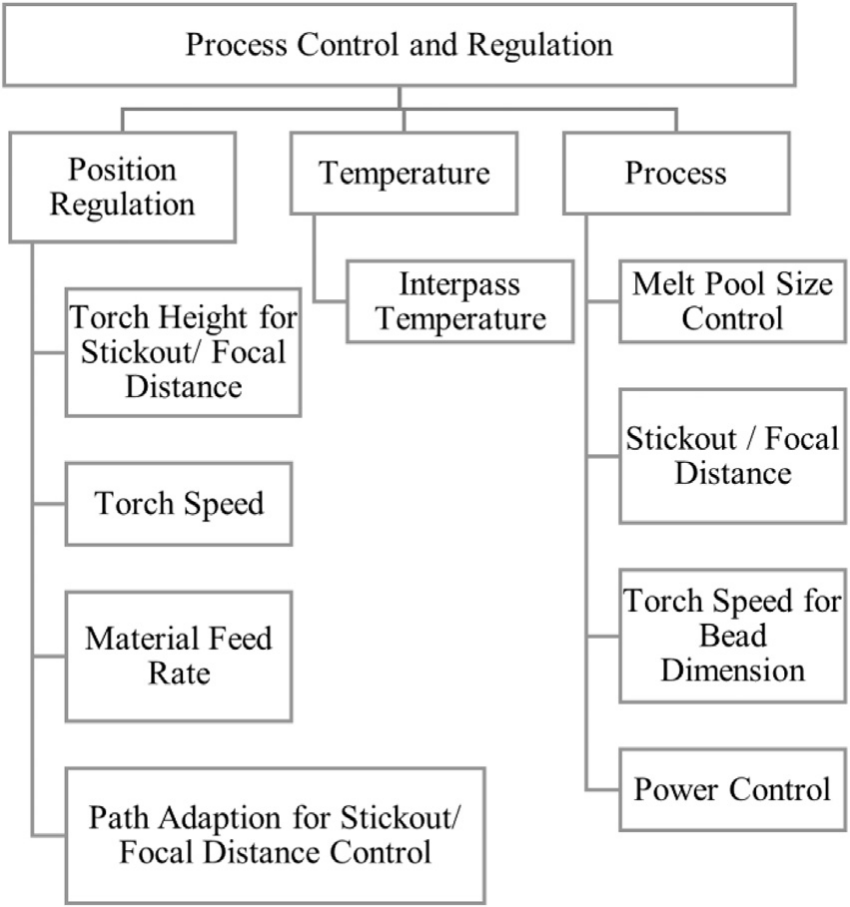
‘Process control and regulation’ influence group.

Process Control and Regulation, Fig. 10, is a very relevant group in the digitized DED-Process. To account for distortion, incorrect bead height or other measured data, the torch may be repositioned, effectively reslicing the workpiece, yielding fundamental reshaping of the surface. Control loops towards interpass temperature, melt pool size, stickout, torch speed and process power have been reported in literature [[3], [4], [5], [6], [7], [8]] and will affect the shape the melt pool has, influencing the surface shape after all.

**Fig. 11:**
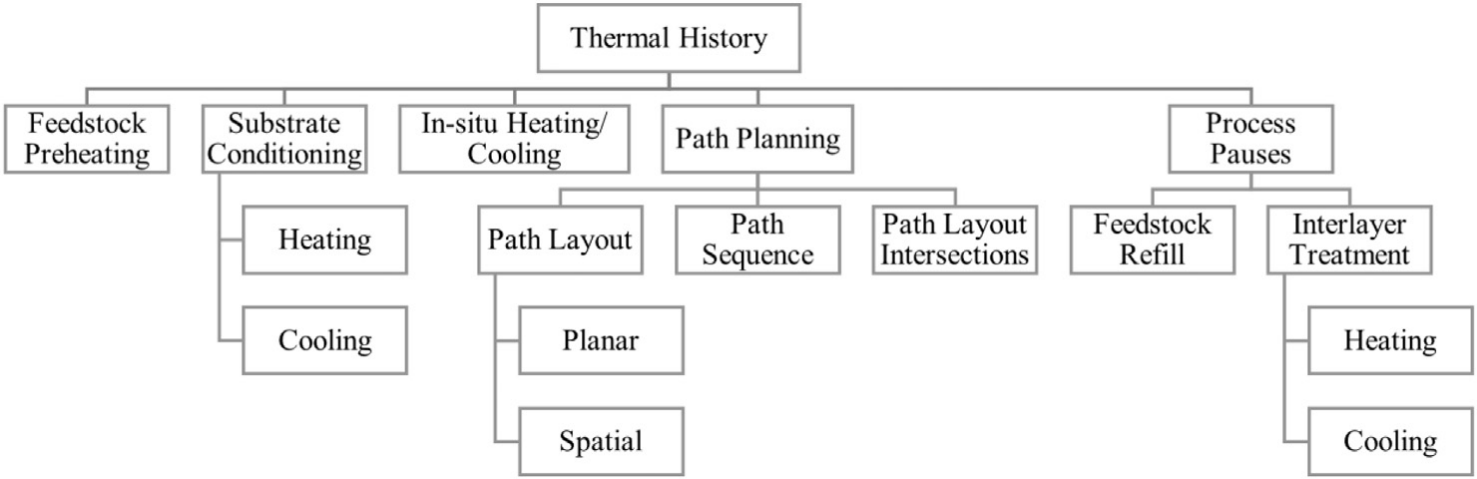
‘Thermal history’ influence group.

The Thermal History, Fig. 11, influences melting of the material deposited by influencing the prerequisites at the working spot. By artificial measures such as feedstock preheating, heating and cooling of substrate or workpiece in process pauses or even in-situ, the base material temperature the bead is placed upon is influenced. But also involuntary factors such as spatial and temporal formation of path planning or process pauses will affect the material temperature immediately before the bead is placed upon it. The temperature will determine melt pool shape, thus influencing surface formation.

**Fig. 12:**
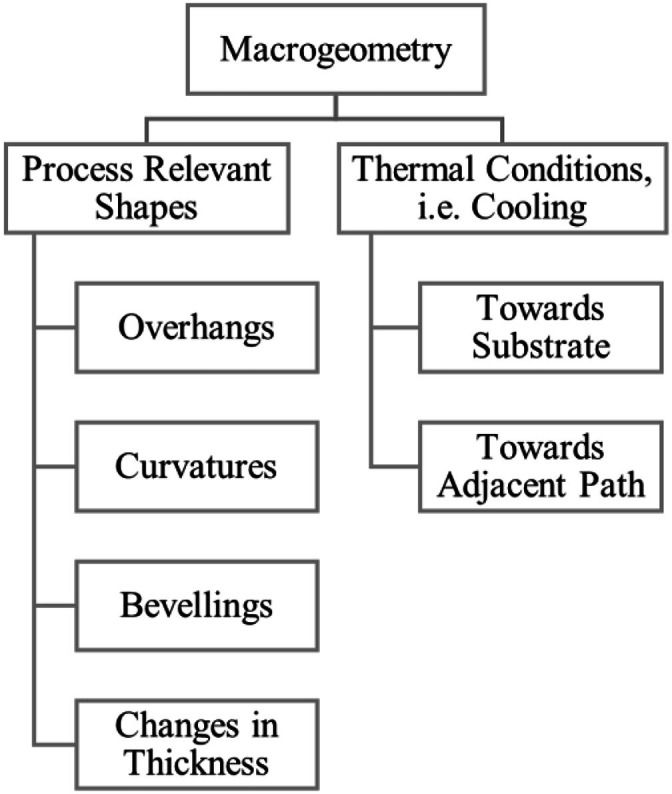
‘Macrogeometry’ influence group.

The workpiece’ macrogeometry, Fig. 12, also has a great effect on the surface formation, as it is the strongest boundary condition on the thermal history. In addition to that, the workpiece will also affect bead shape by direct geometry effects. Overhangs will affect the shape the melt pool solidifies in as well as other geometry shapes such as thickness changes. Moreover, the macroscopic shape is having a direct effect on cooling of the deposited bead, as it determines the number of paths the heat can be distributed to. Geometrically abrupt changes in heat flow paths, for instance at thickness changes, separations or joint points of bifurcartions will again influence the solidification of the molten pool, affecting surface formation.

**Fig. 13:**
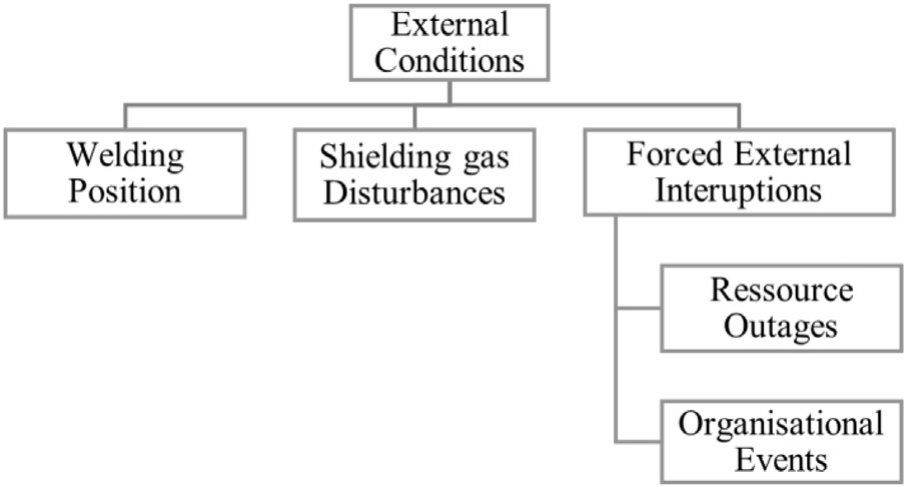
‘External conditions’ influence group.

External conditions, Fig. 13, to the process need to be considered as well. Most prominent is the welding position on the workpiece relative to gravity, again shaping the melt pool. External events, such as power, personnel or feedstock outages as well disturbances of shielding gas by outages or effects like wind or housing/covering failures are not related to the process in the first place, but affect thermal conditions and melt pool shape.

## Limitations

‘Not applicable’.

## Ethics Statement

The authors confirm that the they have read and followed the ethical requirements for publication in Data in Brief and confirm that the current work does not involve human subjects, animal experiments, or any data collected from social media platforms.

## CRediT Author Statement

Jonny Kaars: Conceptualization, Data Curation, Formal Analysis, Investigation, Methodology, Project Administration, Resources, Software, Validation, Visualization, Writing – Original Draft; Jonas Hensel: Conceptualization, Data Curation, Funding Acquisition, Project Administration, Resources, Supervision, Validation, Writing – review & editing

## Data Availability

Mendeley DataDED-Surface Feature Sample Catalogue (Original data)
Mendeley DataInfluence factors on the surface structure of DED-workpieces (Original data)
